# Haspin regulates Ras localization to promote Cdc24-driven mitotic depolarization

**DOI:** 10.1038/s41421-020-0170-2

**Published:** 2020-06-23

**Authors:** Roberto Quadri, Martina Galli, Elena Galati, Giuseppe Rotondo, Guido Roberto Gallo, Davide Panigada, Paolo Plevani, Marco Muzi-Falconi

**Affiliations:** 1 Dipartimento di Bioscienze, Università degli Studi di Milano, Via Celoria 26, 20133 Milano, Italy; 2 Present Address: IFOM, Istituto FIRC di Oncologia Molecolare, Via Adamello 16, 20139 Milano, Italy

**Keywords:** Cell polarity, Mitosis

## Abstract

Cell polarization is of paramount importance for proliferation, differentiation, development, and it is altered during carcinogenesis. Polarization is a reversible process controlled by positive and negative feedback loops. How polarized factors are redistributed is not fully understood and is the focus of this work. In *Saccharomyces cerevisiae*, mutants defective in haspin kinase exhibit stably polarized landmarks and are sensitive to mitotic delays. Here, we report a new critical role for haspin in polarisome dispersion; failure to redistribute polarity factors, in turn, leads to nuclear segregation defects and cell lethality. We identified a mitotic role for GTP-Ras in regulating the local activation of the Cdc42 GTPase, resulting in its dispersal from the bud tip to a homogeneous distribution over the plasma membrane. GTP-Ras2 physically interacts with Cdc24 regulateing its mitotic distribution. Haspin is shown to promote a mitotic shift from a bud tip-favored to a homogenous PM fusion of Ras-containing vesicles. In absence of haspin, active Ras is not redistributed from the bud tip; Cdc24 remains hyperpolarized promoting the activity of Cdc42 at the bud tip, and the polarisome fails to disperse leading to erroneously positioned mitotic spindle, defective nuclear segregation, and cell death after mitotic delays. These findings describe new functions for key factors that modulate cell polarization and mitotic events, critical processes involved in development and tumorigenesis.

## Introduction

Cells of almost all living organisms undergo a phase of polarization, in which material deposition and cell growth are directed towards specific areas of the cell periphery. Understanding the mechanisms overseeing this process is of pivotal importance as its deregulation can lead to severe diseases and is one of the first steps of malignant transformation in carcinogenesis^1^. Indeed, one of the first steps of the epithelial to mesenchymal transition (EMT), a process that provides cancer cells with the capability to outnumber the surrounding tissues and to move and invade distal compartments of the organism^2^, is the loss, or alteration, of cellular polarization.

A family of small proteins, Rho GTPases, oversees cellular polarity, with the protein Cdc42 playing a major role from budding yeast to human cells^3^. In *Saccharomyces cerevisiae* early G1 cells, Cdc42 promotes symmetry breaking to generate a bud from an otherwise round cell. Initially, GTP-bound Cdc42 forms a polar cap and, after bud emergence, the clustered activity of Cdc42 at the bud tip directs growth of the daughter cell manipulating the actin cytoskeleton. At the end of mitosis, Cdc42 activity drops to allow cytokinesis^4^.

The activity of Cdc42 is regulated by GTPase-activating proteins (GAPs), guanine nucleotide exchange factors (GEFs), and guanosine nucleotide dissociation inhibitors (GDIs). The budding yeast genome codes for a single GDI, Rdi1, and four GAPs (Rga1, Rga2, Bem2, and Bem3^5–8^). The main GEF for Cdc42 in this organism is the essential protein Cdc24, which orchestrates the activation of GTP-Cdc42 in differentially localized clusters during the cell cycle^9,10^, and is an absolute prerequisite for *S.cerevisiae* cells to bud^11^. A second GEF has been recently reported, which is important for polarity establishment in early G1^12^. In late G1, Cdc24 localizes at the presumptive bud-site and then, from S to M-phase, it accumulates at sites of polarized growth; it is then sequestered into the nucleus during late M-phase until the next budding^13,14^. Recruitment of Cdc24 to the plasma membrane (PM) in G1 relies on its physical interaction with Rsr1, a Ras-family GTPase, and with the scaffold protein Bem1; deletion of either one does not prevent budding, while loss of both is lethal. Interestingly, some *rsr1∆bem1∆* cells survive and are still able to polarize to some extent, suggesting the existence of yet another player that can promote clustering of active Cdc42^15–17^.

Work in other organisms suggested the existence of a physical interaction between Cdc24 and active-Ras^18,19^. Ras GTPases are ubiquitous in eukaryotic cells, where they play a fundamental role in cell cycle regulation, indeed Ras signaling is altered in several types of human cancers^20^. In budding yeast the main role of Ras paralogues, Ras1 and Ras2, is to regulate cell cycle commitment in G1 by activating protein kinase A (PKA), in response to external factors^21,22^. Ras exerts its essential role upon accumulation at the PM, which is achieved through a secretory apparatus-dependent and a secretion-independent pathway^23–26^. The activity of Ras in *S.cerevisiae* is modulated by two GAPs (Ira1 and Ira2) and two GEFs, the essential Cdc25, and the dispensable Sdc25, which only takes part in Ras activation upon growth on poor media^27–33^. Beside its essential role in G1, Ras can also participate to mitosis in budding yeast and other organisms^34–37^.

The physiological significance of the interaction between Ras and Cdc24 and the mechanistic details underlying a role of Ras in regulating Cdc42 have not been investigated in detail^18,19^.

The atypical protein-kinase haspin is conserved in eukaryotes, suggesting that it may play an important function in the cell cycle. Previous reports indicate that haspin is recruited at centromeric regions in a topoisomerase II-dependent manner^38,39^. There, haspin phosphorylates threonine 3 of histone H3 (H3-Thr3) and promotes the recruitment of the chromosome passenger complex (CPC) and efficient chromosome segregation^40–47^. In budding yeast, two haspin paralogues, Alk1 and Alk2, have been identified^48^, and we have shown that they play an essential role in tolerating M-phase delays, such as those induced by nocodazole or by delayed activation of APC/Cdc20^49^. Indeed, when mitosis is delayed, loss of haspin activity causes the missegregation of both nuclei to the daughter cell and cell death. This phenotype is accompanied by a strong hyper-accumulation of actin in the enlarged bud^49^. We suggested that an altered regulation of polarization, either as increased polarization or as failure to disperse polarized factors, may be responsible for these phenotypes^49^.

While the establishment of polarization has been widely studied, the mechanisms underlying its dispersal and the consequences of depolarization failure have not been investigated in detail. Recent data showed that Cdc24 is subjected to Cdc28-dependent and Cla4-dependent phosphorylation, which alters its dynamics at the PM, suggesting that phosphorylation is a mechanism to disperse Cdc24 from the bud tip^50^.

In this work, we analyzed the involvement of *S. cerevisiae* haspin in polarization dispersal. We show that yeast haspin ultimately regulates the dispersal of Cdc42 activity, the master player of polarization. This function is exerted by modulating the recruitment of Cdc24, the main Cdc42 GEF. We report that Cdc24 localization is regulated by Ras and that haspin promotes a change in Ras-loaded vesicle fusion, shifting it from a preferentially bud tip-directed to a uniform fusion to the daughter PM. Haspin activity thus is responsible for restoring a uniform distribution of Cdc24 and dispersing the bud tip cluster of active Cdc42.

## Results

### Defective distribution of active Cdc42 in haspin mutants leads to hyperpolarization

In budding yeast cells experiencing a mitotic delay, loss of haspin leads to the accumulation of actin and nuclear missegregation within the daughter cell^49^. We showed this to be the consequence of a persistent accumulation of polarity factors at the bud tip^49^, possibly due to defective redistribution of polarized clusters.

**Fig. 1:**
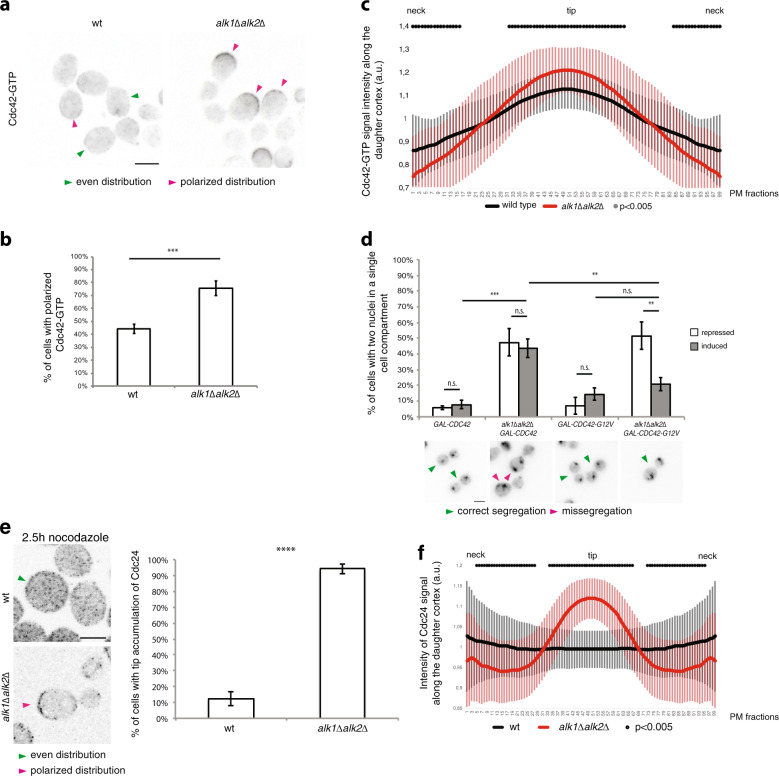
Haspin modulates the distribution of Cdc42-GTP and Cdc24. **a**–**c** Localization of active Cdc42 was assessed through a CRIB-TdTomato probe, specific for Cdc42-GTP. G1-arrested wt or haspin-lacking cells were released in nocodazole-containing medium for 2.5 h and analyzed by fluorescence microscopy **a**, scoring the percentage of cells with polarized Cdc42-GTP **b**. Green and magenta arrows in panel **a** show cells with isotropic distribution of GTP-Cdc42 and sites of polarized Cdc42 activity, respectively; scale bar: 5 μm. **c** Average CRIB-tomato signal intensity along the daughter cell PM (see section “Materials and methods” for full description). **d** wt or hyperactive Cdc42, under the control of the GAL1 promoter, was expressed for 45 min in mitotically arrested control or *alk1Δalk2Δ* cells. The effect of Cdc42 overexpression on nuclear segregation localization was assessed by fluorescence microscopy after an M-phase delay. Green and magenta arrows point at: correct or defective segregation. **e**, **f** After pre-synchronization in G1, wild-type or haspin-lacking cells were released into nocodazole-containing medium. The localization of Cdc24-GFP was evaluated by fluorescence microscopy. Cells that accumulate the GEF at the bud tip were scored at given time points (green and magenta arrows indicate examples of cells with diffuse PM localized Cdc24 and daughters with polarized Cdc24, respectively); graphs report the percentage of cells with Cdc24 at the bud tip scored 2.5 h after G1 release in nocodazole-containing medium; error bars represent standard deviation. Cdc24-GFP signal intensity along the PM quantified on 60 nocodazole-arrested cells from three independent experiments is reported in **f**. 3 **d** or 4 **b** independent experiments were performed, counting 100 cells for each repeat; error bars represent standard deviation. Scalebars correspond to 5 μm. *t*-test was applied as a statistical measurement in **b**, **d**, **e**; n.s.: not significant; *P < 0.05; **P < 0.01; ***P < 0.005; ****P < 0.001. Black dots in **c**–**f** represent fractions for which the intensity between the strains is significantly different (*P* < 0.005, calculated with Bonferroni correction); error bars represent standard deviation.

The principal regulator of polarity in eukaryotes is the GTPase Cdc42. To characterize the impact of haspin on active Cdc42 in mitotic cells, we analyzed the mitotic localization of its GTP-bound form, using a chimeric CRIB-TdTomato fluorescent probe that specifically binds to GTP-loaded Cdc42^4,51,52^. Following nocodazole treatment, exploited here to induce a mitotic delay, in the majority of control wt cells the Cdc42-GTP fluorescence signal is distributed homogeneously, while only 44% of the cells exhibit a polarized distribution (Fig. 1a–c and Supplementary Fig. S1a). In the absence of haspin, on the other hand, active Cdc42 is largely found at the bud tips (76% polarized cells; Fig. 1a–c). This observation was supported by measuring the distance between the geometric center of the cell (centroid, which is linked to the cell shape) and the fluorescence center of mass (that measures the distribution of fluorescence intensity); this parameter accounts for discrepancies from a uniform distribution of fluorescence^53,54^. Consistent with the rest of the data, this value is significantly higher in *alk1∆alk2∆* cells compared to wt counterparts (Supplementary Fig. S1b).

To verify whether the defective localization of active-Cdc42 was responsible for the phenotypes of *alk1∆alk2∆* mutants, we tried to force a more uniform distribution of Cdc42 activity by overexpressing the GTPase in nocodazole-arrested wt or haspin-lacking cells. As shown in Supplementary Fig. S1c, even following *CDC42* overexpression, haspin mutants did not restore a homogeneous distribution of Cdc42-GTP and active Cdc42 was still found mostly at the bud tip. On the contrary, overexpression of a constitutively active *CDC42-G12V* allele, which does not need Cdc24 for activation, led to a more uniform distribution of Cdc42-GTP in *alk1∆ alk2∆ cells* (Supplementary Fig. S1c)^55^. Both alleles were expressed at similar levels (Supplementary Fig. S1e) and did not significantly affect cell cycle profiles of the host cells (Supplementary Fig. S1d). We also found that while increased expression of wt *CDC42* did not suppress the abnormal nuclear segregation observed in *alk1∆alk2∆* strains, overexpression of Cdc42-G12V suppresses nuclear the missegregation phenotype of haspin-lacking cells. To exclude possible artefacts, we show that, while overexpression of constitutively active *CDC42* perturbed the morphology of logarithmically growing cells (Supplementary. S1f), as previously reported^55^, it did not lead to hyperpolarization in nocodazole-arrested cells (Fig. 1d and see “Discussion” section).

Cdc42 is involved in shmoo formation following exposure to mating pheromones. We can exclude that haspin plays a significant role during α-factor treatment exploited to achieve G1-arrested populations in our experiments. As reported in Nespoli et al. (2006), Alk1 and Alk2 are not expressed in G1 cells and are actively degraded. Moreover, as shown in our FACS analyses, both strains responded similarly to mating pheromone. Finally, loss of haspin did not change the percentage of cells with polarized Cdc42-GTP in G1 (Supplementary Fig. S1g).

Altogether, these results suggest that loss of haspin results in the mitotic confinement of active Cdc42 to the bud tip, where it promotes the mislocalization of key polarity factors and, ultimately, nuclear missegregation and cell death and that defective redistribution of active-Cdc42 is responsible for the hyperpolarization of haspin mutants.

### Haspin regulates mitotic Cdc24 localization

Cdc42 activation in budding yeast mainly relies on the essential GEF Cdc24^5–10^: precise localization of Cdc24 is crucial to locally activate Cdc42. The accumulation of polarized Cdc42-GTP at the bud tip might be explained by a persistent polarization of the GEF. We thus analyzed Cdc24 localization in wt and haspin-lacking cells.

wt and *alk1Δalk2Δ* cells expressing Cdc24-GFP were pre-synchronized in G1 and released in nocodazole-containing medium; 2.5 h after the release, we monitored the localization of the GEF in mitotic cells (Fig. 1e, f). In most control cells, Cdc24 is homogeneously diffused, consistently with the uniform distribution of active Cdc42 at the cortex. In contrast, in the absence of haspin Cdc24 accumulates at the bud tip (note that, in agreement with our previous report^49^, in mitotically delayed haspin mutants daughter cells are generally larger then mother cells), explaining the elevated levels of active Cdc42 GTPase at the same location. This finding was confirmed measuring the centroid-center of mass distance, as described above (Supplementary Fig. S1h).

Cdc24 has been recently shown to be the target of a series of mitotic phosphorylation by Cdc28 and Cla4 and that these PTMs regulate its redistribution from the bud tip to the whole cortex^50^. We excluded that the defective redistribution of Cdc24 observed in *alk1∆alk2∆* cells may be caused by defective Cdc24 phosphorylation. Indeed, a phosphomimetic (Cdc24-28D) mutant exhibited a clear polarization in mitotically arrested haspin-lacking cells, similarly to the wt and phosphomutant (Cdc24-46A) forms (Supplementary Fig. S1i). This result clearly suggested that in haspin mutants the cause for Cdc24 misdistribution resided in other factors acting as scaffolds for its localization.

Cdc24 localization is strictly regulated in the different phases of the cell-cycle. In G1, initial accumulation of Cdc24 at the presumptive bud site is promoted by Rsr1 and Bem1, while the factors involved in its mitotic distribution are currently unknown^15^. A role for Rsr1 and Bem1 in mitosis is unlikely. We arrested in nocodazole *rsr1∆ bem1∆* cells complemented by either wt *BEM1* or a ts *bem1-8* allele. A 45 min incubation at non-permissive temperature, is sufficient to inactivate bem1-8 and express the typical polarization defective phenotype in logarithmically growing cells (budded cells: 74% control cells, 30% ts cells). Following inactivation of bem1-8 in mitotically arrested *rsr1∆* cultures, cells did not exhibit any obvious defect in Cdc24-HA-GFP distribution (Supplementary Fig. S1j), proving that a different mechanism must exist to recruit Cdc24 in mitosis.

### Ras-GTP and Cdc24 physically interact

A physical interaction between Cdc24 and active Ras has been previously described in *S. pombe* and *C. neoformans*, although its functional significance was not determined^18,19^.

**Fig. 2:**
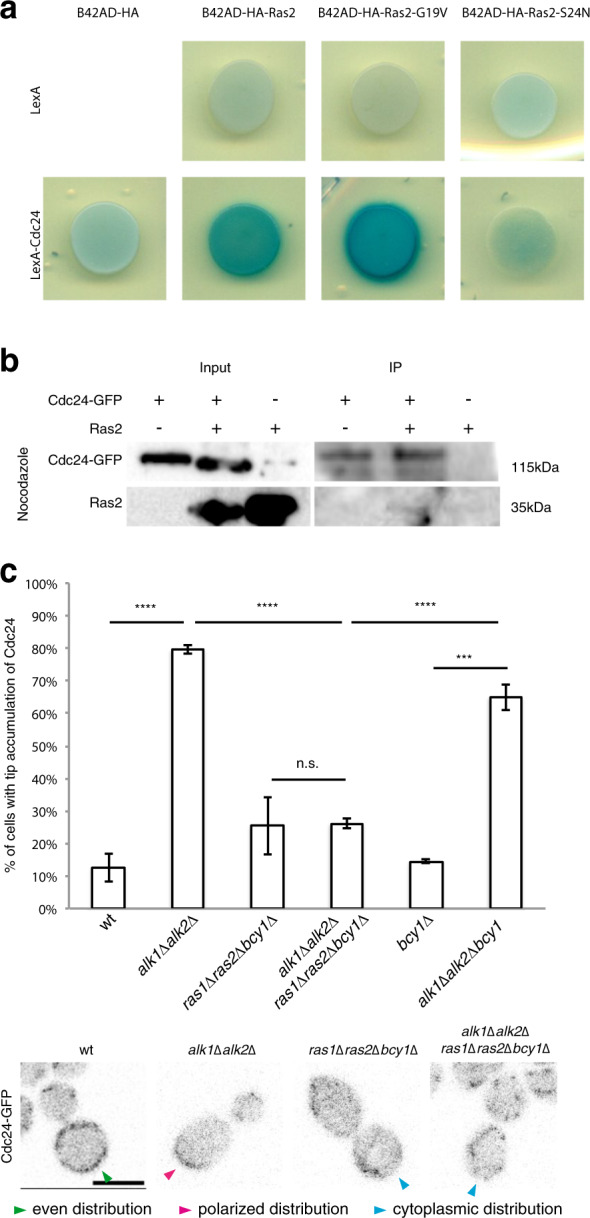
Direct binding to Ras positions mitotic Cdc24. **a** Two-hybrid. EGY48 cells, carrying LacZ under control of LexAop, were transformed with the indicated bait and prey constructs (described in “Materials and methods” section). Isolated transformants were patched on selective plates containing raffinose, galactose, to induce the preys, and X-Gal to detect β-gal activity; blue staining is indicative of physical interaction between fusion proteins. **b** Cells of given strains were arrested in nocodazole. Immunoprecipitation of Cdc24-GFP was conducted as described in “Materials and methods” section and immunoblot analysis with antibodies against GFP or Ras2 performed. The plot in **c** represents the percentage of cells with polarized GFP-Cdc24 in the indicated strains after 3 h of nocodazole treatment. No substantial differences in GFP-Cdc24 expression levels were detected (Supplementary Fig. S2b). Five (panel **c**) independent experiments were performed, counting 100 cells for each repeat. Green, magenta, and cyan arrows show cells with even PM Cdc24 distribution, sites of polarized GEF and cells with cytoplasmic Cdc24, respectively. Scale bars in c: 5 μm. *t*-test was applied as a statistical measurement in **c**; n.s.: not significant; *P < 0.05; **P < 0.01; ***P < 0.005; ****P < 0.001.

To test whether Ras might be involved in recruiting Cdc24 during mitosis in budding yeast, we examined a possible physical interaction between Cdc24 and Ras2 by two-hybrid analyses. We employed a Cdc24 bait and three different Ras2 preys: a wt, a constitutively active (Ras2-G19V) and a dominant-negative (Ras2-S24N) form of Ras2^56,57^. As shown in Fig. 2a, Cdc24 interacts with wt and with constitutively active Ras2, while it fails to interact with dominant negative Ras2; β-galactosidase quantifications are reported in Supplementary Fig. S2a. To further confirm the interaction between Cdc24 and Ras2 through biochemical means, we performed co-immunoprecipitation of GFP-Cdc24 and endogenous Ras2. As shown in Fig. 2b and Supplementary Fig. S2b, recovery of Cdc24-GFP from a crude cell extract led to a concomitant precipitation of Ras2 from both nocodazole-arrested and logarithmically growing cell, demonstrating that the two proteins interact. These results suggest that Cdc24 interacts specifically with GTP-loaded Ras2 also in budding yeast and led us to investigate the contribution of Ras to mitotic Cdc24 localization.

### Ras is required for proper mitotic Cdc24 distribution and resistance to mitotic delays

The budding yeast genome encodes for two Ras paralogues, Ras1 and Ras2. Viable cells carrying the double deletion can be obtained by removing Bcy1, the inhibitory subunit of PKA. Deletion of *BCY1* does not alter the distribution of Cdc24 in nocodazole-arrested wt or *alk1∆alk2∆* cells (Fig. 2c).

To verify whether Ras played any role in the localization of Cdc24, we deleted *RAS1* and *RAS2* in wt or haspin lacking strains. Deletion of *RAS1* and *RAS2* in wt cells leads to a reduction in the overall population of cortex-bound GEF (Supplementary Fig. S2c), although a small fraction of cells with polarized Cdc24 persists (Fig. 2c; as shown in Supplementary Fig. S2d, no evident effect on Cdc24-GFP levels in Ras mutants were detected). These findings suggest that Ras normally modulates the mitotic recruitment of Cdc24 to the PM. We noticed that a basal level of Cdc24 polarized at the bud tip does not depend upon Ras for its localization. In the absence of haspin, when the fraction of cells exhibiting bud-tip polarized Cdc24 is greatly increased, removal of Ras leads to the mitotic displacement of this additional population of hyperpolarized Cdc24, bringing it down to the basal level observed in *ras1∆ras2∆bcy1∆* cells. In the absence of haspin, a larger Ras-dependent accumulation of Cdc24 at the bud tip is observed. These observations suggest that Ras is involved in directing Cdc24 to the PM, leading to the expectation that Ras mutations may functionally affect cell polarity and nuclear segregation in cells subjected to mitotic delays.

**Fig. 3:**
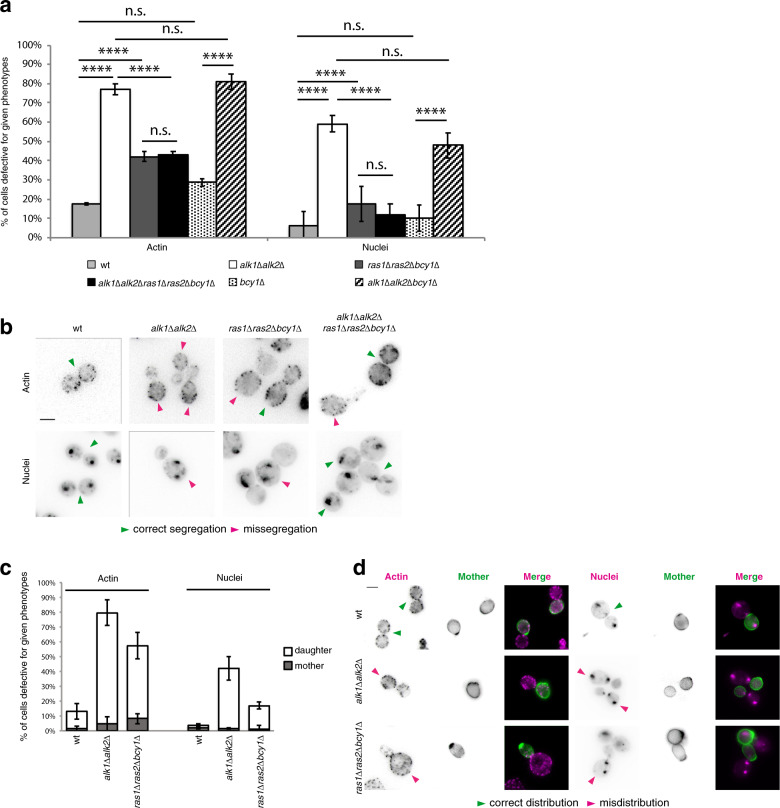
Ras is required for tolerance of M-phase delays. **a**, **b** Cultures of the indicated strains were treated for 3 h with nocodazole. At the end of the treatment, the drug was washed out and actin distribution (*t* = 0 min from the release) and nuclear segregation (*t* = 60 min from the release) were monitored as reported in “Material and methods” section. The percentage of cells exhibiting non-homogeneous actin distribution between mother and daughter cells or missegregated nuclei is reported in **a**; representative images are shown in **b**. **c**, **d** To distinguish mothers from daughters, cells were initially incubated for 10 min with ConA-488 to label the cell wall and then grown without ConA-488 for 1 h to allow cell division. This results in the labeling of the future mothers. Nocodazole was added to the cultures for 3 h. After release in fresh medium, cells were stained for actin and nuclei as in the previous experiment. Actin and nucleus distribution were analyzed by fluorescence microscopy, scoring mitotic cells where only one cell compartment (corresponding to the mother cells) was labeled by ConA-488. For graphs in (**a** and **c**), three independent experiments were performed, counting 100 cells per repeat; error bars represent standard deviation. Scale bars in **b**, **d**: 5 μm. *t*-test was applied as a statistical measurement in **a**, **c**; n.s.: not significant; *P < 0.05; **P < 0.01; ***P < 0.005; ****P < 0.001.

Figure 3a, b, Supplementary Fig. S3 confirm that nocodazole-treated *ras1∆ras2∆bcy1∆* cultures exhibit a fraction of cells that are defective for actin distribution and nuclear segregation, in agreement with the residual bud-tip accumulation of Cdc24 observed in Ras mutants. A peculiarity of haspin mutants is that nuclear missegregation and actin accumulation occur in the daughter cells^49^. Considering that these strains have been reported to display a reduced sensitivity to mating pheromones, to monitor the phenotypes of daughter cells in *ras* mutants, we discriminated mothers from daughters by exploiting fluorophore-conjugated ConcanavaninA, which aspecifically stains the cell wall. Logarithmically growing cells were incubated with ConA to stain all cells. Following a 1 h chase in fresh medium without the dye, the newborn cells remain unstained. Cultures were then arrested in nocodazole and then released in fresh medium without the drug to monitor actin distribution and nuclear segregation at 0 or 60 min after the release, respectively. With this setup we were able to obtain mitotic cells where just the mother was labeled by ConAl. As shown in Fig. 3c, d, this experiment allowed us to confirm that in *ras* mutants actin and nuclei accumulate mostly in daughters, similarly to what happens upon haspin loss.

In *alk1∆alk2∆* cells, where Ras is responsible for the accumulation of Cdc24 at the bud tip, deletion of *RAS1 RAS2* largely restored actin distribution and nuclear segregation to that of a *ras1∆ras2∆* background, suppressing the phenotypes due to loss of haspin (Fig. 3a, b).

Altogether, these findings identify Ras as a critical factor for the recruitment of Cdc24 to the PM and for its redistribution from the bud tip during mitosis. They also suggest that haspin may modulate the distribution of Ras thus facilitating the dispersal of the bud-tip bound fraction of Cdc24. When haspin is present, Ras-dependent redirection of Cdc24 to the cell cortex would promote Cdc24 delocalization from the polarity cap. In the absence of haspin, Ras mislocalization would lead to the large accumulation of extra Cdc24 at the bud tip and to hyperpolarization.

### Haspin regulates localization of Ras

**Fig. 4:**
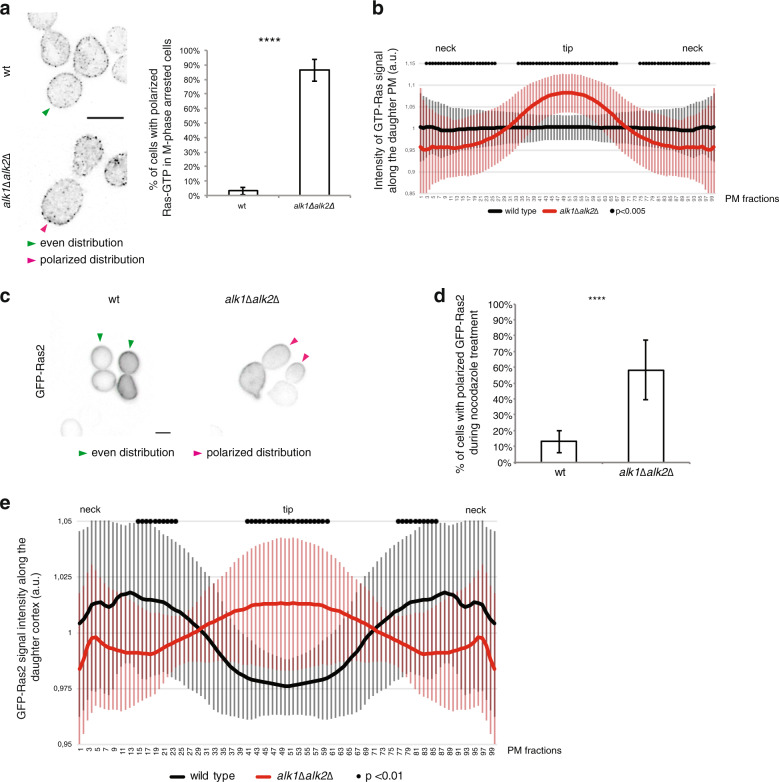
Haspin regulates Ras distribution. The localization of Ras-GTP in wild-type or haspin-lacking cells was evaluated by fluorescence microscopy exploiting an eGFP-RBD3 probe **a** and **b**. Graph in **a** reports the percentage of cells with polarized active-Ras during nocodazole treatment; plot in **b** shows the average intensity of Ras-GTP in mitotic cells. **c**–**e** Cells were arrested in G1 and then released for 2.5 h in nocodazole. Galactose was added at the beginning of nocodazole treatment to induce expression of *GFP-RAS2*. At the end of the treatment cells were fixed and the percentage of cells with polarized Ras2 were scored by fluorescence microscopy. Panel **c** shows a representative image; graph **d** shows the percentage of polarized cells; plot in **e** reports the average intensity of Ras2-GFP in mitotically arrested cells. Green and magenta arrows in **a** and **c** show cells with diffuse PM or polarized given proteins, respectively. Plots in **b** and **e** show the fluorescence intensity signal of given constructs along the PM from 60 cells from three independent experiments (black dots represent fractions for which the intensity between the strains is significantly different (*P* < 0.01, calculated using Bonferroni correction); error bars represent standard deviation). *t*-test was applied as a statistical measurement in (**a**, **b**, **d**, **e**); n.s.: not significant; *P < 0.05; **P < 0.01; ***P < 0.005; ****P < 0.001.

To clarify how haspin impacts on active Ras and hence modulates Cdc24 distribution, we investigated whether haspin affected the localization of Ras-GTP. We monitored active Ras localization using an eGFP-RBD3 probe. The specificity for GTP-loaded Ras was reported in the literature^58–60^ and was further confirmed by verifying that the PM GFP signal observed in wt cells, is maintained in *rsr1∆* but not in *ras1∆ras2∆bcy1∆* mutants (Supplementary Fig. S4a). We report that loss of haspin leads to a preferential localization of active Ras towards the bud tip, following a mitotic delay. In wt cells, on the other hand, GTP-Ras is homogenously distributed over the PM of both mother and daughter cells (Fig. 4a, b, Supplementary Fig. S4b, c).

To understand the relationship between haspin and active Ras, we analyzed Ras regulators and Ras localization in *alk1∆ alk2∆* cells.

Ras activity is modulated by two GEFs, Cdc25 and Sdc25, and two GAPs, Ira1 and Ira2^27–33^. Since Sdc25 is known to be active only in particular nutrient conditions, we tested the possibility that mislocalization of Cdc25 may explain the altered distribution of active-Ras in haspin-defective cells^33^. As shown in Supplementary Fig. S4d, Cdc25 accumulates at internal structures, corresponding to the nucleus and ER membranes, as previously reported^26,61^; we found no differences in its distribution in wt or *alk1∆alk2∆* cells during an M-phase delay (Supplementary Fig. S4d). Along the same lines, we excluded a role for Ira1 and Ira2 in the formation of a hyperpolarized population of active Ras. Removal of Ras GAPs, in fact, has no significant effect on the phenotypes of *alk1∆alk2∆* cells (Supplementary Fig. S4e). If haspin does not modulate local Ras activation, we hypothesized that it may control the localization of the global pool of Ras protein. Analysis of localization of GFP-Ras2 during a nocodazole treatment confirmed that deletion of *ALK1* and *ALK2* caused a generalized reduction of Ras from the PM, leaving a preferential residual accumulation of Ras2 protein at the bud tip. On the contrary, in wt cells with normal haspin activity Ras2 was homogenously distributed on the PM (Fig. 4c–e and Supplementary Fig. S4f).

Overall, these results demonstrate that haspin is a critical factor to ensure a proper dispersal of Ras activity through protein relocalization. In the absence of haspin, failure to redistribute Ras-GTP leads to persistent accumulation of Cdc24, Cdc42-GTP at the bud tip, when anaphase onset is delayed.

### Haspin promotes isotropic vesicle-mediated Ras distribution to the PM during mitosis

Localization of Ras to the PM in budding yeast relies on two distinct pathways, an Erf2/Erf4-dependent mechanism that promotes Ras palmitoylation, and a secretion-dependent mechanism based on vesicular traffic. Loss of either one of the two branches of Ras localization does not prevent PM recruitment of the GTPase, while abrogation of both results in accumulation of the GTPase on endomembranes^24,25^.

**Fig. 5:**
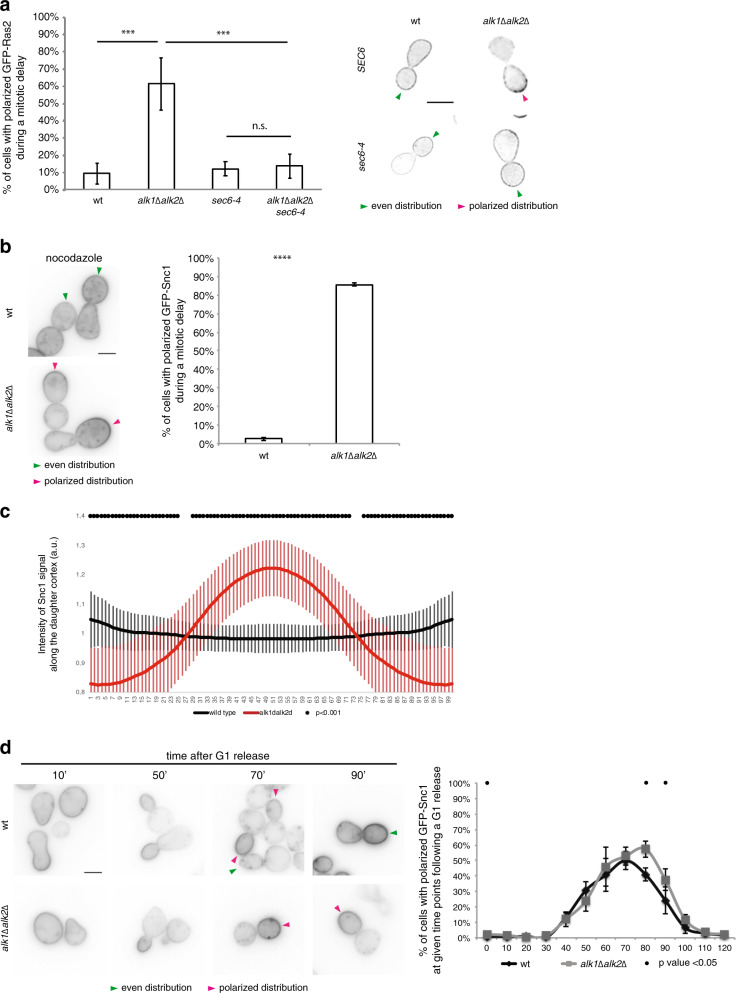
Haspin promotes a shift from apical- to whole PM-oriented vesicle delivery required for Ras dispersion. **a** Cells of the indicated strains were grown at permissive temperature (25 °C) in raffinose-containing medium. After G1-synchronization, strains were released in nocodazole containing medium supplemented with 2% galactose to induce expression of GFP-*RAS2*. After 2.5 h, cultures were shifted to 37 °C to inactivate exocytosis for further 1.5 h. Samples were then taken and analyzed by fluorescence microscopy. The graph represents the percentage of cells with polarized GFP-Ras2; representative exhibits are shown on the right, green and magenta arrows indicate cells with even or polarized GFP-Ras2 signal, respectively. wt or haspin-lacking cells expressing GFP-SNC1 were arrested in G1 and then released for 2.5 h in nocodazole-containing medium **b** and **c** or released in fresh medium without drugs **d**. Samples were analyzed by fluorescence microscopy. Representative images are shown, and the quantified results are reported in the graphs. Green and magenta arrows indicate cells with even or polarized Snc1, respectively. For graphs **a**, **b**, **d** three experiments were performed counting 100 cells per repeat, error bars represent standard deviation. Cdc24-GFP signal intensity along the PM quantified on 60 nocodazole-arrested cells from three experiments were performed, counting 100 cells for each repeat; error bars represent standard deviation. Scale bars in **a**, **b**, **d**: 5 μm. *t*-test was applied as a statistical measurement in **a**–**d**; n.s.: not significant; *P < 0.05; **P < 0.01; ***P < 0.005; ****P < 0.001.

We tested whether defective distribution of Ras in haspin mutants stems from preferential vesicle fusion to the bud tip. We generated wt or *alk1∆alk2∆* strains expressing *GFP-RAS2* in a temperature-sensitive *sec6-4* genetic background. At restrictive temperature, the conditional allele of the Sec6 SNARE impairs vesicle tethering to the PM blocking the vesicle-mediated pathway of Ras targeting to the PM, without affecting its recruitment through the Erf2-mediated pathway. If loss of haspin led to Ras accumulation at the bud tip due to preferential vesicular fusion, the *sec6-4* mutation should rescue Ras hyperpolarization in *alk1∆alk2∆* cells. Inactivation of Sec6 completely abolished the uneven accumulation of Ras in *alk1∆alk2∆* cells, while it did not affect the homogeneous PM distribution of Ras2 in otherwise wt cells (Fig. 5a and Supplementary Fig. S5a). These data demonstrate that haspin is needed for isotropic Ras redistribution along the cortex through vesicles, and that, in the absence of haspin, the of Ras-containing vesicles preferentially fuse in the bud tip area leading to the hyperpolarization observed in *alk1∆ alk2∆* cells.

We then monitored vesicles routes in wt and haspin-lacking cells using GFP-tagged Snc1 (a v-SNARE) to label vesicles. Cells were arrested in G1 and then released in the presence of nocodazole. We report that loss of haspin caused a persistent enrichment of Snc1-labeled vesicles at the bud tip-proximal region of the PM, following a M-phase arrest (Fig. 5b, quantified in Fig. 5c and Supplementary Fig. S5b). In *alk1∆ alk2∆* cells, some polarity factors are mislocalized also in unperturbed cell cycle^49^; we thus expect haspin mutants to exhibit a more persistent bud tip localization of Snc1 also in an unperturbed mitosis. This was confirmed by monitoring GFP-Snc1 localization in a G1-to-G1 cell cycle analysis (Fig. 5d and Supplementary Fig. S5c). To rule out the possibility that the phenotypes observed in haspin mutants may stem from slight delays in cell-cycle progression, we exploited a conditional *mif2-3* strain grown at a semi-permissive temperature. In these conditions, cell cycle progression is noticeably delayed, yet we did not detect increased Snc1 polarization (Supplementary Fig. S5d).

Deletion of *RAS1 RAS2* did not prevent the accumulation of Snc1-labeled vesicles at the tip in haspin mutants (Supplementary Fig. S5e), while it suppressed the hyperpolarization of Cdc24 (Fig. 2c) and the defective actin distribution (Fig. 3a, b). These observations clearly indicate that Ras acts downstream of vesicle fusion and upstream of Cdc24 in the axis between haspin and polarisome dispersal.

Haspin exerts its functions through its kinase activity and, up to now, no kinase-independent roles have been identified. To verify whether the kinase activity of Alk1 and Alk2 was responsible for the role of haspin in the pathway described herein, we employed *ALK1* and *ALK2* alleles carrying partially inactivating mutations in the catalytic site described previously^62^. We analyzed the distribution of GFP-Snc1 in mitotically arrested cells. As shown in Supplementary Fig. S5f, such mutants are partially defective for GFP-Snc1 distribution, indicating a requirement of the kinase activity of Alk1 and Alk2 for depolarization in budding yeast.

Overall, we describe how haspin kinase, regulating exocytosis of Ras-containing vesicles, controls a novel regulatory axis that promotes the dispersal of polarization-promoting factors. In budding yeast this pathway is critical to tolerate mitotic delays and we predict that it may be significantly relevant also in higher organisms.

## Discussion

Control of cell polarity is critical for organism development, organ and tissue function and differentiation. Polarity alterations are linked to pathologies and carcinogenesis, making understanding the bases for polarity regulation a key challenge. Budding yeast *S. cerevisiae* has proven to be an invaluable tool to dissect polarity onset and the function of the small GTPase Cdc42 along with its positive (GEF) or negative (GAPs and GDIs) regulators. Studies in this organism, indeed, provided a wealth of information on how polarization is established and maintained to allow proper cell growth. We still lack, however, a complete picture of how cells deal with polarity dispersion and what are the consequences of a failure in such process. Previously, we provided a first insight on the effects of a prolonged polarization on budding yeast cells. We reported that *alk1∆alk2∆* cells, lacking haspin kinase, accumulate excessive polarity factors at the bud tip. Following an M-phase delay, induced by transient spindle depolymerization (nocodazole treatment) or due to ineffective APC (repression of *CDC20* expression), this hyperpolarization causes actin accumulation in the daughter cell, nuclear missegregation and ultimately cell death^49^. Here, we reveal a new role for haspin in polarisome dispersion. Haspin activity drives redistribution of Ras along the PM, leading to delocalization of Cdc24 and consequently of Cdc42 activity. Our results demonstrate that timely relocalization of polarity factors is a fundamental event in the cell cycle.

We previously reported an abnormal polarization in haspin-lacking cells and we proposed this to be the leading cause for actin and nuclear segregation defects in such cells. The small GTPase Cdc42 regulates polarization in all eukaryotes, and impairments in Cdc42 result in non-properly organized actin networks in budding yeast and human cells, making it an appealing candidate for haspin-dependent regulation^63,64^.

In the absence of haspin, GTP-Cdc42 accumulates at the bud tip, compared to a diffused distribution in wt control cells. Overexpression of a constitutively active but not a wt *CDC42* led to a redistribution of Cdc42 activity over the PM and alleviated the phenotypes imposed by haspin loss. This suggests that the problem in haspin-lacking cells is not the distribution of the Cdc42 protein, but rather its local activation. We thus analyzed the distribution of Cdc24, the main GEF responsible for activating Cdc42. In control mitotic cells, Cdc24 was dispersed all over the cell membrane, reflecting the homogenous distribution of GTP-Cdc42. Conversely, in the absence of haspin Cdc24 was mostly found at the bud tip, explaining the accumulation of GTP-Cdc42 in the same region.

It was thus essential to determine how Cdc24 is redistributed from the bud tip homogeneously to the PM. At the beginning of the cell cycle, Cdc24 localization at the incipient bud site is established through the interaction with the Ras-family protein Rsr1 and Bem1, however concomitant loss of Rsr1 and Bem1 had no impact on Cdc24 localization in mitotic cells, suggesting the existence of distinct, cell cycle-specific recruitment mechanisms for the GEF.

Previous reports suggested the existence of a physical interaction between Cdc24 and Ras in other organisms^18,19^. Through CoIP and two-hybrid analyses, we demonstrated an in vivo physical interaction between GTP-Ras2 and Cdc24 in budding yeast, suggesting a possible mechanism for modulating Cdc24 redistribution.

We confirmed that the dispersal of polarized Cdc24 in mitotic cells is controlled by RAS. Deletion of *RAS1 RAS2* in *alk1∆alk2∆* cells suppressed the persistence of Cdc24 at the bud tip, the actin unbalance and nuclear missegregation caused by loss of haspin.

Works by Yoshida et al. and Geymonat et al. showed that Lte1, which initially accumulates at the bud tip, is recruited during mitosis to the PM through the binding to GTP-Ras^35,65^. The switch in Lte1 interactors is promoted by phosphorylation events mediated by Cdc28 and the PAK Cla4. Intriguingly, phosphorylation of mitotic Cdc24 by the same kinases was recently reported to be critical for its redistribution from the bud tip to the whole PM^50^. This supports a bipartite model for Cdc24 recruitment during the cell cycle^15,50^. In the early stages, when polarized growth is needed, Rsr1 and Bem1 cooperatively promote the accumulation of non-phosphorylated Cdc24 at the bud tip. Accordingly, *rsr1∆bem1∆* mutants are inviable (with few background-specific and laboratory-specific exceptions) due to budding defects. We suggest that later in the cell cycle, when cells switch to a phase of isotropic growth, phosphorylation of Cdc24 may promote its Ras-dependent homogeneous redistribution to the PM, leading to its dispersal from the bud tip. Ras could play a role as a distributed scaffold to relocalize phosphorylated Cdc24 and hence Cdc42 activity all over the PM in the different phases of the cell-cycle, when polarity clusters have to be resolved.

The Ras-dependent redistribution of Cdc24 is linked to the localization of Ras itself rather than to its local activation, consistently with the notion that Ras activation takes place in the endomembrane compartment^26,61^. Indeed, we show that, while normally Ras is found homogenously distributed on the PM, in *alk1∆ alk2∆* cells it is mostly localized at the bud tip. Exploring how haspin may modulate Ras localization, we found that loss of haspin leads to alterations of the exocytic pathway and to the preferentially fusion of Ras-containing vesicles at the bud tip. All such phenotypes are likely caused by the loss of haspin kinase activity since mutants in the catalytic site exhibit a phenotype resembling that of deleted strains. We ascribe the lower incidence of the phenotype in such mutants to the residual kinase activity retained by the cells (described in our previous work^62^).

**Fig. 6:**
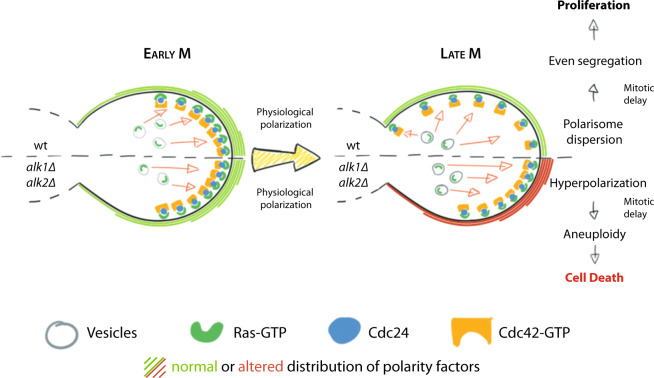
Graphic summary. Haspin-dependent isotropic fusion of Ras-containing-vesicles on the PM is essential for a successful mitosis. During M-phase, Cdc24 relies on GTP-Ras to be recruited to the PM, possibly through direct physical interaction. This relocalization of Cdc24 promotes an even distribution of Cdc42 activity and hence polarisome dispersal. In early mitosis, the fusion of vesicles that mediate Ras-GTP delivery to the PM occurs mainly at the bud tip, causing accumulation of Cdc24 and subsequently of GTP-Cdc42 to the same region. As the cell cycle progresses through late mitosis, wt cells direct homogeneous vesicle fusion to the whole PM causing a delocalization of GTP-Ras, Cdc24, and active Cdc42 from the bud tip. Loss of haspin impairs this shift in vesicle fusion, causing a persistent polarized traffic towards the bud tip with consequent persistency of the polarity factors. This persistent polarization, in case of mitotic delays, ultimately results in defective nuclear segregation and consequent cell death.

Haspin is a cell cycle regulated kinase, active mostly in mitosis. We show that following cell polarization, haspin is important for a uniform vesicle fusion to the PM leading to a diffused distribution of GTP-loaded Ras. This in turn leads to the dispersal of Cdc24 from the bud-tip and its relocalization to the cell cortex through physical interactions. The shift in the pattern of Cdc24 leads to the redistribution of GTP-Cdc42 as schematically represented in Fig. 6. The actual step regulated by haspin is still unknown; we however hypothesize that the kinase might modulate the distribution of cortical spatial landmarks regulating vesicle fusion, such as the membrane-bound Sec3 and Exo70 subunits of the exocyst complex, whose localization is driven by phosphatidylinositol accumulation to different sites of the membrane. This aspect will be the topic of future works.

This process is essential to tolerate M-phase delays. This work provides, to the best of our knowledge, the first mechanistic insight on how the depolarization process is promoted and reports novel functions for haspin and Ras.

Given the extreme conservation of the proteins involved, we propose that this regulatory pathway may be conserved in all eukaryotes to regulate polarization-driven processes that are often altered in carcinogenesis.

## Materials and methods

### Yeast strains and plasmids

All strains and plasmids used in this study are listed in Tables 1 and 2. Standard conditions for yeast cell cultures have been previously described^66^. Standard molecular genetics techniques were used to construct plasmids and strains. The centromeric plasmids containing *GFP-3RBD, CDC25-eGFP*, and *GST-RBD* were kind gifts of Dr. E. Martegani^60^, the one coding for *GST* was kindly provided by Dr. D. Pellman. *GFP-BUD6* and *CDC24-eGFP*-bearing strains were obtained transforming cells with pRB2190 and pYS37, respectively^67,68^. CRIB-TdTomato and *rsr1∆bem1∆* strains were kindly provided by Dr. D.J. Lew^50,52^. Plasmids and strains encoding Cdc24 phosphosite mutants were a kind gift of Dr. D. McCusker^50^. PCR-based genotyping was used to confirm gene disruption and tagging. Gene overexpression or repression with *GAL1* promoter were achieved by adding 2% galactose or 2% glucose, respectively, to raffinose-containing medium.

**Table 1:** Strains used in this work.

Name	Relevant genotype	Source
K699	*ade2-1 trp1-1 can1-100 leu2-3,112 his3-11,15 ura3 MATa*	K.Nasmyth
*BF264-15D	*ade1 his2 leu2-3,112 trp1-1 ura3∆ns*	B.Futcher^73^
**EGY48	*ura3 his3 trp1 6xLexAop-LEU2 MATα*	R.Brent
yRQ301	*CRIB-TdTomato-KANr MATa*	This work
yRQ302	*alk1::NATr alk2::HIS3 CRIB-TdTomato-KANr MATa*	This work
yRQ500	*CRIB-TdTomato-KANr [pGAL-CDC42] MATa*	This work
yRQ501	*CRIB-TdTomato-KANr [pGAL-CDC42-G12V] MATa*	This work
yRQ502	*alk1::NATr alk2::HIS3 CRIB-TdTomato-KANr [pGAL-CDC42] MATa*	This work
yRQ503	*alk1::NATr alk2::HIS3 CRIB-TdTomato-KANr [pGAL-CDC42-G12V] MATa*	This work
yRQ100	*[CDC24-GFP] MATa*	This work
yRQ101	*alk1::NATr alk2::HIS3 [CDC24-GFP] MATa*	This work
yRQ451	*[CDC24-mEOS] MATa*	This work
yRQ452	*[CDC24-46A-mEOS] MATa*	This work
yRQ453	*[CDC24-28D-mEOS] MATa*	This work
yRQ454	*alk1::NATr alk2::HIS3 [CDC24-mEOS] MATa*	This work
yRQ455	*alk1::NATr alk2::HIS3 [CDC24-46A-mEOS] MATa*	This work
yRQ456	*alk1::NATr alk2::HIS3 [CDC24-28D-mEOS] MATa*	This work
*yRQ505	*rsr1::HIS3 bem1::KANr [BEM1] [CDC24-HA-GFP] MATa*	This work
*yRQ506	*rsr1::HIS3 bem1::KANr [bem1-8] [CDC24-HA-GFP] MATa*	This work
yRQ366	*bcy1::KANr [CDC24-GFP] MATa*	This work
yRQ367	*alk1::NATr alk2::HIS3 bcy1::KANr [CDC24-GFP] MATa*	This work
yRQ368	*ras1::TRP1 ras2::HPHr bcy1::KANr [CDC24-GFP] MATa*	This work
yRQ369	*alk1::NATr alk2::HIS3 ras1::TRP1 ras2::HPHr bcy1::KANr [CDC24-GFP] MATa*	This work
yAN33	*alk1::NATr alk2::HIS3 MATa*	This work
yRQ358	*ras1::TRP1 ras2::HPHr bcy1::KANr MATa*	This work
yRQ359	*alk1::NATr alk2::HIS3 ras1::TRP1 ras2::HPHr bcy1::KANr MATa*	This work
**yRQ479	*[pADH-LexA-CDC24] [pGAL1-B42AD-HA] [LexAop-LacZ] MATα*	This work
**yRQ480	*[pADH-LexA] [pGAL1-B42AD-HA-RAS2] [LexAop-LacZ] MATα*	This work
**yRQ481	*[pADH-LexA] [pGAL1-B42AD-HA-RAS2-G19V] [LexAop-LacZ] MATα*	This work
**yRQ490	*[pADH-LexA] [pGAL1-B42AD-HA-RAS2-S24N] [LexAop-LacZ] MATα*	This work
**yRQ483	*[pADH-LexA-CDC24] [pGAL1-B42AD-HA-RAS2] [LexAop-LacZ] MATα*	This work
**yRQ484	*[pADH-LexA-CDC24] [pGAL1-B42AD-HA-RAS2-G19V] [LexAop-LacZ] MATα*	This work
**yRQ491	*[pADH-LexA-CDC24] [pGAL1-B42AD-HA-RAS2-S24N] [LexAop-LacZ] MATα*	This work
YRQ102	*ras2::TRP1 [CDC-24-GFP] MATa*	This work
yRQ73	*[eEGFP-RBD3] MATa*	This work
yRQ74	*alk1::NATr alk2::HIS3 [eGFP-RBD3] MATa*	This work
yRQ442	*ras1::TRP1 ras2::HPHr bcy1::KANr [eGFP-RBD3] MATa*	This work
yRQ472	*rsr1::KANr [eGFP-RBD3] MATa*	This work
yRQ84	*[CDC25-GFP] MATa*	This work
yRQ85	*alk1::NATr alk2::HIS3 [CDC25-GFP] MATa*	This work
yRQ93	*ira1::LEU2 ira2::URA3 MATa*	This work
yRQ95	*alk1::NATr alk2::HIS3 ira1::LEU2 ira2::URA3 MATa*	This work
yRQ395	*[pGAL-GFP-RAS2] MATa*	This work
yRQ396	*alk1::NATr alk2::HIS3 [pGAL-GFP-RAS2] MATa*	This work
yRQ492	*sec6-4 [pGAL-GFP-RAS2] MATa*	This work
yRQ493	*alk1::NATr alk2::HIS3 sec6-4 [pGAL-GFP-RAS2] MATa*	This work
yRQ197	*[GFP-SNC1] MATa*	This work
yRQ198	*alk1::NATr alk2::HIS3 [GFP-SNC1] MATa*	This work
yRQ478	*mif2-3 [GFP-SNC1] MATa*	This work
yRQ479	*alk1::NATr alk2::HIS3 mif2-3 [GFP-SNC1] MATa*	This work
yRQ444	*ras1::TRP1 ras2::HPHr bcy1::KANr [GFP-SNC1] MATa*	This work
yRQ445	*alk1::NATr alk2::HIS3 ras1::TRP1 ras2::HPHr bcy1::KANr [GFP-SNC1] MATa*	This work
yRQ538	*Alk1*^*KD*^*::URA3 Alk2*^*KD*^*::URA3 [GFP-SNC1] MATa*	This work

All strains used are isogenic to W303 apart from those carrying deletions of *RSR1* and *BEM1*, which are isogenic to BF264-15DU (marked with * in the table) and those used in two hybrid assays which are isogenic to EGY48 (marked with**).

**Table 2:** Plasmids used in this work.

Name	Relevant genotype	Source
pRQ24	*pRS314-pGAL1-CDC42*	This work
pRQ25	*pRS314-pGAL1-CDC42-G12V*	This work
pYS37	*pRS315-CDC24-GFP*	M. Peter^68^
pDM700	*pRS416-pCYC1-CDC24-mEOS-HIS6*	D. McCusker^50^
pDM701	*pRS416-pCYC1-CDC24-46A-mEOS-HIS6*	D. McCusker^50^
pDM704	*pRS416-pCYC1-CDC24-28D-mEOS-HIS6*	D. McCusker^50^
p414Cdc24HAGFP	*pRS314-CDC24-HA-GFP*	R. Arkowitz^13^
pSH18-34	*8xLexAop-LacZ*	R. Brent
pEG202	*pADH-LexA*	R. Brent
pJG4-5	*pGAL1-B42AD-HA*	R. Brent
pRQ35	*pJG4-5-RAS2*	This work
pRQ36	*pJG4-5-RAS2-G19V*	This work
pRQ37	*pJG4-5-RAS2-S24N*	This work
pRQ38	*pEG202-CDC24*	This work
PB1622	*pGEX-5×-1-GST*	D. Pellman^4^
pGEX2T-RBD	*pLac-GST-RBD*	E. Martegani^59^
pYX242-eGFP-RBD3	*pYX242-eGFP-3RBD*	E. Martegani^58^
yEPCDC25eGFP	*CDC25-GFP*	E. Martegani^74^
B828	*yEP55-RAS2-GFP*	R.J. Deschenes^75^
GFP-SNC1	*pRS315-pTPI1-GFP-SNC1*	K. Tanaka^76^

### Western blotting

To analyze proteins during nocodazole treatment, cells were grown in YPD medium, synchronized in G1 with α-factor (2 μg/ml), and released in the presence of nocodazole (10 μg/ml). At given time points, samples were collected to obtain total protein extracts that were resolved by SDS–PAGE and analyzed by western blotting using proper antibodies (A-6455 for GFP, Ab6160 for tubulin, 22c5d8 for Pgk1, sc-6759 for Ras2), as previously described^69^. Images were taken with a ChemidocTouch Imaging System (Bio-Rad) and processed with ImageLab and ImageJ^70^.

### Image quantification and statistics

Cells were synchronized as previously described, fixed with formaldehyde (3.7%) and washed three times in PBS^49^.

Localization was determined with a Leica DMRA2 widefield fluorescence microscope or a Nikon A1 N-SIM system; images were processed with FIJI^70^. The percentage of cells with polarized proteins was determined by counting cells with a preferential accumulation of signal at the bud tip; experiments were performed at least once in blind fashion.

To evaluate the asymmetric distribution of a fluorescent label, we employed a system where the position of the geometrical center of the cell (centroid) is compared to the position of the center of fluorescence mass signal. If the fluorescence signal is equally distributed, the centroid and the fluorescence center of mass are superimposed. If the fluorescence is polarized, the fluorescence mass center is shifted with respect to the centroid. The distance between centroid and fluorescence mass center is an indication of the degree of polarization^53,54^. The centroid to center of mass distance was calculated on 60 cells per strain using ImageJ and normalized to the daughter cell area and circularity; statistical significance was determined with a *t*-test (see Supplementary Fig. S6a).

Signal intensity on the cell membrane was quantified as follows. Fluorescence intensity on the cortex of 60 daughter cells from three independent experiments was measured. Each cell was divided in 100 parts of the same length, and their intensity was normalized to the total fluorescence of the cell. The average intensity of each fraction was calculated as the mean of normalized fractions from all cells using the following equation, where *I*, *i*, *j*, *n* and *m* represent the intensity, the fraction, the cell, the number of analyzed daughters and the number of fractions, respectively (for further details see Supplementary Fig. S6b, c).

$$\bar I_i = \bar I_{m + 1 - i} = \frac{{\mathop {\sum }\nolimits_{j = 1}^n \frac{{I_{i,j} + I_{m + 1 - i,j}}}{{2\mathop {\sum }\nolimits_{i = 1}^m I_{i,j}}}}}{n}$$

To determine the membrane/cytoplasm ratio of Cdc24, ROIs were traced around 60 cell membranes per strain, and the area and intensity of the ROIs were measured with ImageJ. The cytoplasm intensity was determined eroding the ROIs by five pixels and normalizing the raw intensity on the area. The differences in intensity and area between outer ROI and cytoplasm were used to calculate the PM intensity.

Statistical analyses were performed with *t*-test and results expressed using following abbreviations: n.s. not significant; *P < 0.05; **P < 0.01; ***P < 0.005; ****P < 0.001.

### Cell-cycle synchronization

Cells were synchronized in either G1 or mitosis exploiting α-factor or nocodazole as follows. For G1 synchronization, cells were grown in required media and incubated with 2 µg/ml α-factor for 2 h (when grown in glucose-containing medium) or 2 h and 15 min (when grown in raffinose-containing medium). To obtain mitotically arrested cells, cultures were incubated for 2.5 h with 10 µg/ml nocodazole. In both cases cell-cycle arrests were monitored by FACS analysis.

### Two-hybrid

EGY48 cells were transformed with the relevant plasmids (pEG202, pJG4-5, and their derivatives expressing fusions with Cdc24, Ras2 alleles). Proper expression of the fusion proteins was verified by western blotting. The *lacZ* reporter is harbored on the pSH18-34 plasmid. p53 and SV40 Large T were used as positive control. Relevant strains were patched on selective raffinose/galactose-containing plates supplemented with 0.195 nM X-Gal, 23.1 mM NaH_2_PO_4_, and 21.1 mM Na_2_HPO_4_. Pictures were taken after overnight incubation at 28 °C. To confirm the physical interaction, a liquid β-galactosidase assay was performed. Briefly, cells were grown in selective liquid medium containing galactose and raffinose as carbon sources. Cultures were pelleted, washed in cold water and mechanically lysed in 250 µl breaking buffer (100 mM Tris–HCl, pH 8.0, 10% glycerol, 1 mM DTT, 1 complete mini (Roche)). Extracts were clarified (10 min 13,000 rpm at 4 °C), protein concentration was determined and 40 µl per sample were incubated at 37 °C with 1 ml of Z-buffer (300 mM NaH_2_PO_4_, 200 mM Na_2_HPO_4_, 50 mM KCl, 5 mM MgSO_4_, 50 mM β-mercaptoethanol, 0.8 mg/ml ONPG). After 15 min from sample addition, reactions were stopped by addition of 400 µl of NaCO_3_ and the OD_420_ was measured. β-galactosidase units were determined using the following equation:

$$U = \frac{{{\mathrm {OD}}_{420} \ast 1,4}}{{0.0045 \ast i \ast v \ast t}}$$

where *i*, *v*, and *t* correspond to input sample concentration (µg/µl), volume of sample added (µl), and reaction time (in s), respectively.

### Co-immunoprecipitation

Analysis of physical interaction by co-immunoprecipitation was performed exploiting a protocol adapted from previous work^71^. 150 ml of nocodazole-arrested cultures were collected, transferred to a screw-cap tube and resuspended in 300 μl IP-buffer (50 mM Tris, pH 7.5, 150 mM NaCl, 1 mM DTT, 10 mM NaF, 50 mM β-glycerolphosphate, 0.1 mM NaVO_4_, 10 mM PNPP, 1 complete tablet EDT A-free (Roche), 1% PMSF, 1% protease inhibitor cocktail (SigmaAldrich), 1% NP-40). Cells were mechanically lysed with glass beads on a vortex at 4 °C, extracts were clarified, transferred to new tubes and normalized to have about 2 mg of proteins in 500 μl of IP-buffer. 50 μl of extracts were incubated with 10 μl of 2x Laemmli buffer and used as INPUT. The remaining volume of extract was incubated with 8 μl of GFP-TRAP^®^_MA for 1 h at 4 °C. Beads were washed two times with IP-buffer and two times with wash buffer (50 mM Tris–HCl, pH 7.5, 150 mM NaCl, 1 mM DTT, 0.1% Triton X-100) before being boiled for 10 min in 50 μl 1x Laemmli buffer.

### Actin staining

Cells were grown as described, fixed with formaldehyde (3.7%), and washed three times with PBS. After incubation for 45 min with Alexa Fluor 594-conjugated phalloidin, actin was visualized by fluorescence microscopy.

### Determination of incorrect anaphase

Cells were synchronized in G1 and released in nocodazole as described above. After 150 min in nocodazole, cells were washed and released in fresh medium without the drug. At the indicated times after removal of nocodazole, cells were fixed with ethanol 100%, washed three times with PBS and stained with DAPI. Cells were then scored by fluorescence microscopy.

### Concanavalin A staining

Exponentially growing cells were washed with PBS and resuspended in 125 μl of Alexa Fluor 488-conjugated concanavalin A (ThermoFisher C11252) at a concentration of 40 μg/ml in the dark at room temperature. After 10 min, cells were washed and resuspended in appropriate medium for 1 h, prior to nocodazole treatment. The initial ConA treatment will stain all cells, both mother and daughters. During the chase period, cell division occurs, and only future mother cells will remain stained. At the end of nocodazole treatment we had three classes of cells: cells with no dye, cells with only one cell compartment stained (specifically the mother, which was present at the time of ConA incubation) and few cells with both compartments stained. We restricted our analysis to those where only a single cell compartment, corresponding to the mother, was stained.

### Cell cycle analysis with FACScan

Samples were taken at given time points, fixed with 70% ethanol and processed with RNase A and Proteinase K, as described in Engels et al.^72^. Cells were then stained with 1 µM SytoxGreen and DNA content was determined using a FACScan cytofluorimeter.

## Supplementary information

Supplementary Figure S1

Supplementary Figure S2

Supplementary Figure S3

Supplementary Figure S4

Supplementary Figure S5

Supplementary Figure S6

## Supplementary information

**Supplementary Information** accompanies the paper at (10.1038/s41421-020-0170-2).
