# Supplementary material for: Haspin regulates Ras localization to promote Cdc24-driven mitotic depolarization
Source: Cell Discov. 2020 Jun 23;6:42. doi: 10.1038/s41421-020-0170-2 (PMC7308332; doi:10.1038/s41421-020-0170-2)
Supplement: Supplementary file 1 — Supplementary Figure S1 [file 41421_2020_170_MOESM1_ESM.pdf]

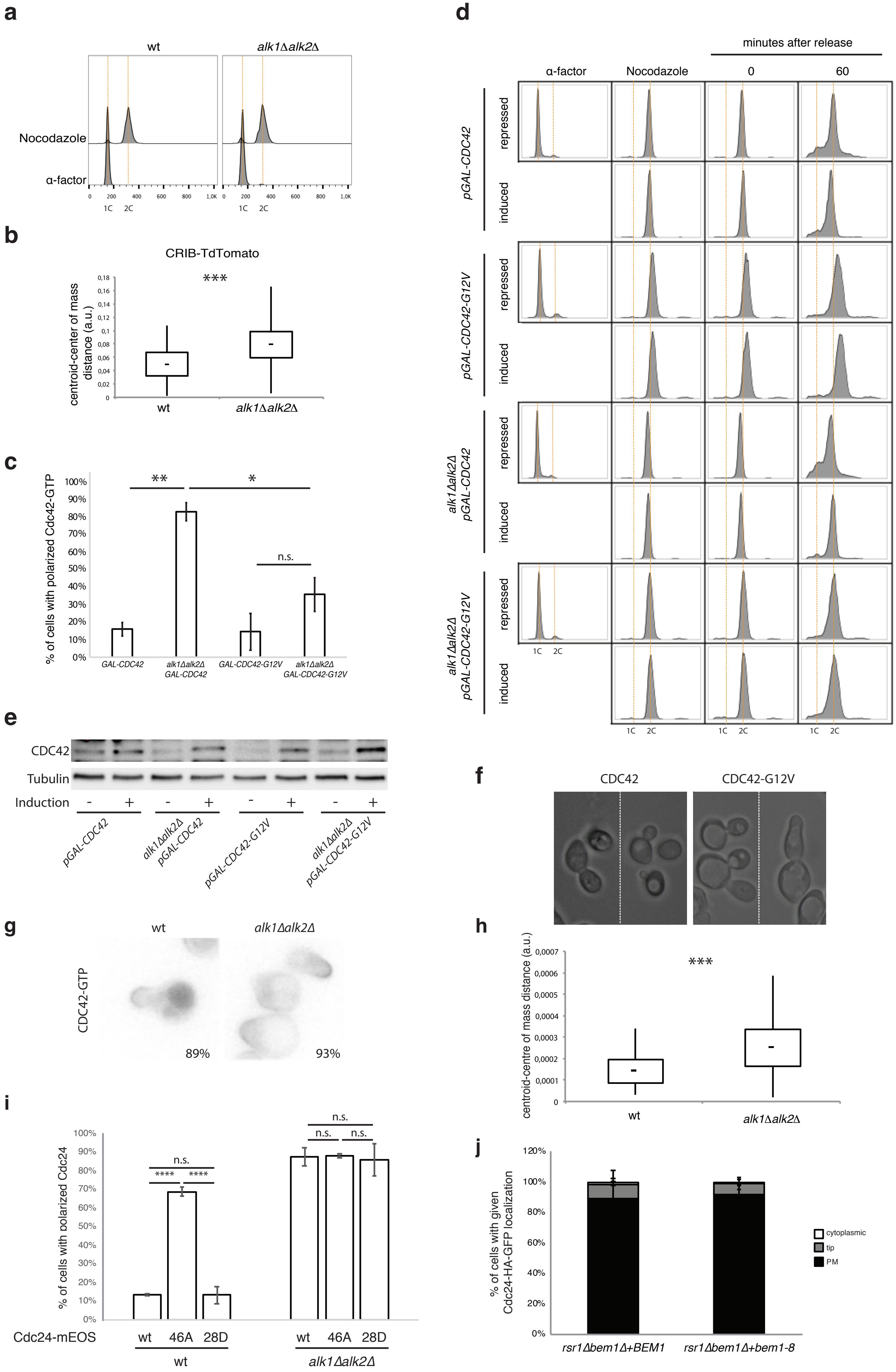
a
wt
alk1Δalk2Δ
Nocodazole
α-factor
1C
2C
b
CRIB-TdTomato
centroid-center of mass distance (a.u.)
***
wt
alk1Δalk2Δ
c
% of cells with polarized Cdc42-GTP
**
*
n.s.
GAL-CDC42
alk1Δalk2Δ GAL-CDC42
GAL-CDC42-G12V
alk1Δalk2Δ GAL-CDC42-G12V
d
minutes after release
α-factor
Nocodazole
0
60
pGAL-CDC42
pGAL-CDC42-G12V
alk1Δalk2Δ pGAL-CDC42
alk1Δalk2Δ pGAL-CDC42-G12V
repressed
induced
e
CDC42
Tubulin
Induction
pGAL-CDC42
alk1Δalk2Δ pGAL-CDC42
pGAL-CDC42-G12V
alk1Δalk2Δ pGAL-CDC42-G12V
f
CDC42
CDC42-G12V
g
wt
alk1Δalk2Δ
CDC42-GTP
89%
93%
h
centroid-centre of mass distance (a.u.)
***
wt
alk1Δalk2Δ
i
% of cells with polarized Cdc24
n.s.
****
Cdc24-mEOS
wt
46A
28D
alk1Δalk2Δ
j
% of cells with given Cdc24-HA-GFP localization
cytoplasmic
tip
PM
rsr1Δbem1Δ+BEM1
rsr1Δbem1Δ+bem1-8

S1. Related to Figure 1.

Panels (a) and (d) report the cell cycle arrests monitored by FACS analysis relative to the experiments in Figs. 1a-c and d, respectively. The plot in (b) represents the centroid-center of fluorescence mass distance of Cdc42-GTP, normalized on the daughter cell area and average daughter cell strain circularity, calculated on 60 cells per strain from 3 independent experiments; boxes include 50% of data points, lines represents the average distance and whiskers report the minimum and maximum values. The graph in (c) reports the percentage of mitotic cells of the indicated strains with polarized active Cdc42. Cells were arrested in mitosis in raffinose-containing medium, overexpression was performed adding 2% galactose for 45 minutes. The experiment was performed twice; error bars represent standard deviation. Panel e: Western blot to control expression of Cdc42 in the experiment in Fig.1 d. (f) morphological aberrations induced by overexpression of constitutively active Cdc42. Logarithmically growing cells were incubated for 1 hours with galactose to induce expression of given Cdc42 alleles and then monitored for cellular morphology (Cdc42-G12V leads to the appearance of multibudded or hyperpolarized cells). Panel g shows the distribution of Cdc42-GTP in G1-synchronized cells, the percentage of cells exhibiting polarized signal is shown. The graph in (h) shows the centroid-center of mass distance of GFP-Cdc24, normalized on daughter area and daughter cell average circularity, of 60 nocodazole arrested cells from 3 independent experiments as in Figure 1e. Boxes include 50% of data points, lines represent the average distance and whiskers report the minimum and maximum values. (i) wt or Haspin-lacking cells expressing given mEOS-tagged Cdc24 alleles were synchronized in nocodazole for 2.5 hours, before monitoring Cdc24-mEOS distribution by fluorescence microscopy. The graph shows the percentage of cells exhibiting polarized accumulation of the GEF from 3 experiments, counting 100 cells per repeat; error bars represent standard deviation. (j) Cells were synchronized in nocodazole for 2 hours 15 minutes at 25°C and further incubated at 37°C for 45 minutes to inactivate bem1-8. Cdc24-HA-GFP localization was analyzed by fluorescence microscopy. The graph reports the percentage of cells of the indicated strains with given Cdc24 distribution from 2 independent experiments. 100 cells were counted per repeat; error bars represent standard deviation. t-test was applied as a statistical measurement in (b,c,g,i); n.s.: not significant; * : p-value <0.05; ** : p-value <0.01; *** : p-value <0.005; **** : p-value <0.001.
