# Supplementary material for: Haspin regulates Ras localization to promote Cdc24-driven mitotic depolarization
Source: Cell Discov. 2020 Jun 23;6:42. doi: 10.1038/s41421-020-0170-2 (PMC7308332; doi:10.1038/s41421-020-0170-2)
Supplement: Supplementary file 2 — Supplementary Figure S2 [file 41421_2020_170_MOESM2_ESM.pdf]

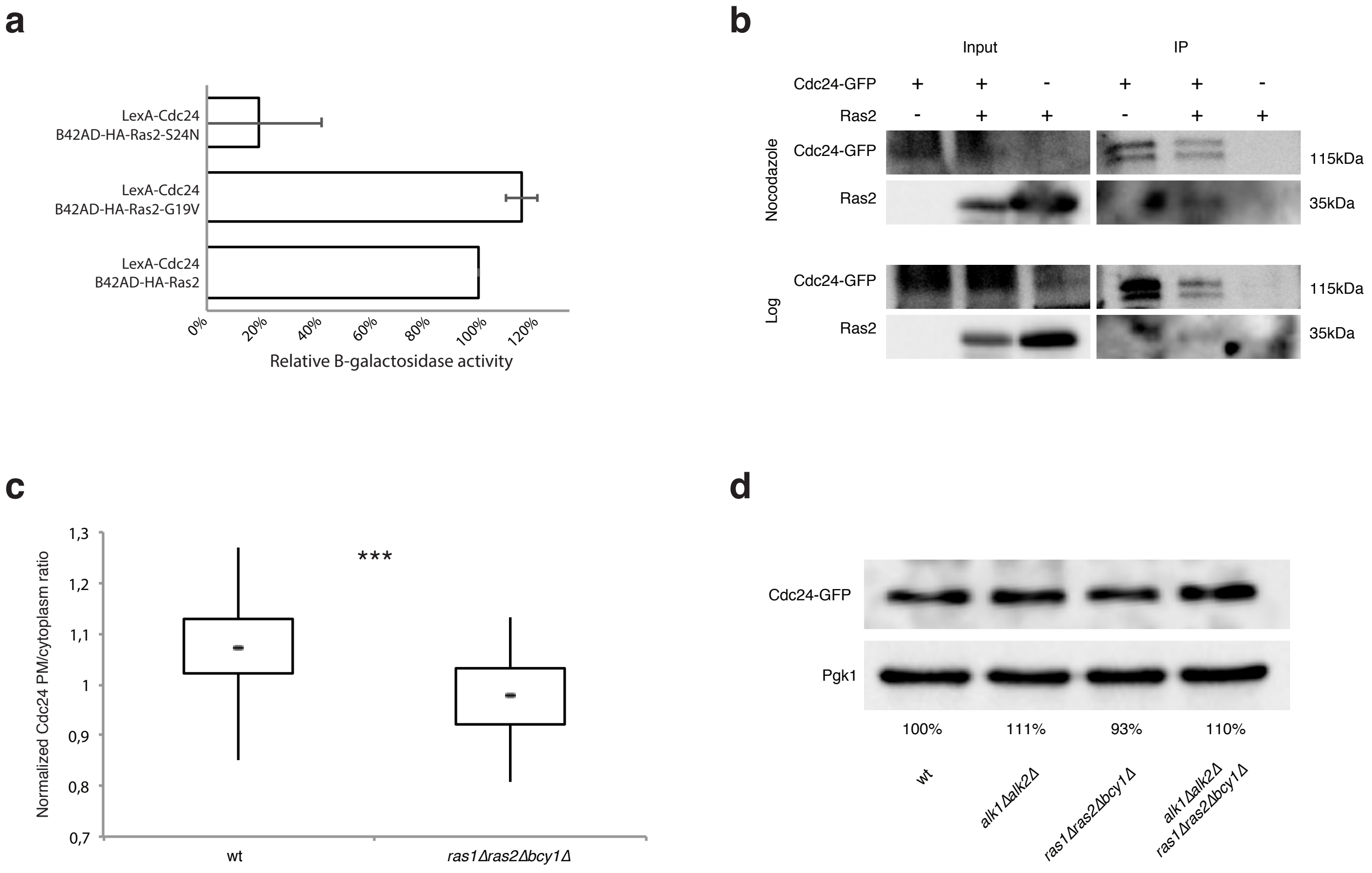

a
LexA-Cdc24
B42AD-HA-Ras2-S24N
LexA-Cdc24
B42AD-HA-Ras2-G19V
LexA-Cdc24
B42AD-HA-Ras2
0%
20%
40%
60%
80%
100%
120%
Relative B-galactosidase activity
b
Input
IP
Cdc24-GFP
Ras2
Nocodazole
Log
115kDa
35kDa
c
Normalized Cdc24 PM/cytoplasm ratio
1,3
1,2
1,1
1
0,9
0,8
0,7
***
wt
ras1Δras2Δbcy1Δ
d
Cdc24-GFP
Pgk1
100%
111%
93%
110%
wt
alk1Δalk2Δ
ras1Δras2Δbcy1Δ
alk1Δalk2Δ
ras1Δras2Δbcy1Δ

S2. Related to Figure 2.

(a) Liquid β-galactosidase assay was performed. Briefly, cells were grown in selective liquid medium containing galactose and raffinose as carbon sources. Samples were collected and processed as described in material and methods to obtain the relative β-galactosidase units. (b) Cells of given strains were either arrested in nocodazole or grown in logarithmically phase. Immunoprecipitation of Cdc24-GFP was conducted as described in material and methods and immunoblot analysis with antibodies against GFP or Ras2 performed. The graph in (c) represents the ratio of normalized Cdc24-GFP fluorescence intensity at the PM and that in the cytoplasm (see material and methods for further details). wt and *RAS*-deleted cells were analyzed by fluorescence microscopy after 3 hours of nocodazole treatment. 60 cells from 3 independent experiments were analyzed. Boxes include 50% of data points, lines represent the average distance and whiskers report the minimum and maximum values. Expression levels of Cdc24-GFP in the different strains employed for the experiment reported in Fig. 2d. Error bars represent standard deviation. t-test was applied as a statistical measurement in (c); n.s.: not significant; * : p-value <0.05; ** : p-value <0.01; *** : p-value <0.005; **** : p-value <0.001.
