# Supplementary material for: Haspin regulates Ras localization to promote Cdc24-driven mitotic depolarization
Source: Cell Discov. 2020 Jun 23;6:42. doi: 10.1038/s41421-020-0170-2 (PMC7308332; doi:10.1038/s41421-020-0170-2)
Supplement: Supplementary file 3 — Supplementary Figure S3 [file 41421_2020_170_MOESM3_ESM.pdf]

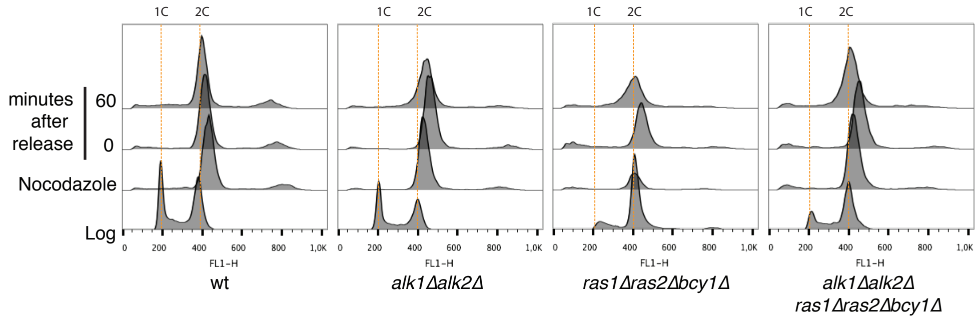
1C
2C
minutes after release
60
0
Nocodazole
Log
0
200
400
600
800
1,0K
FL1-H
wt
alk1Δalk2Δ
ras1Δras2Δbcy1Δ
alk1Δalk2Δ ras1Δras2Δbcy1Δ

S3. Related to Figure 3.

Cell-cycle analysis by FACS of experiment in Figs. 3a-b. Cells were synchronized in G2/M phase by 3 hours nocodazole treatment. 1C and 2C refer to the amount of DNA in the cells.
