# Supplementary material for: Haspin regulates Ras localization to promote Cdc24-driven mitotic depolarization
Source: Cell Discov. 2020 Jun 23;6:42. doi: 10.1038/s41421-020-0170-2 (PMC7308332; doi:10.1038/s41421-020-0170-2)
Supplement: Supplementary file 4 — Supplementary Figure S4 [file 41421_2020_170_MOESM4_ESM.pdf]

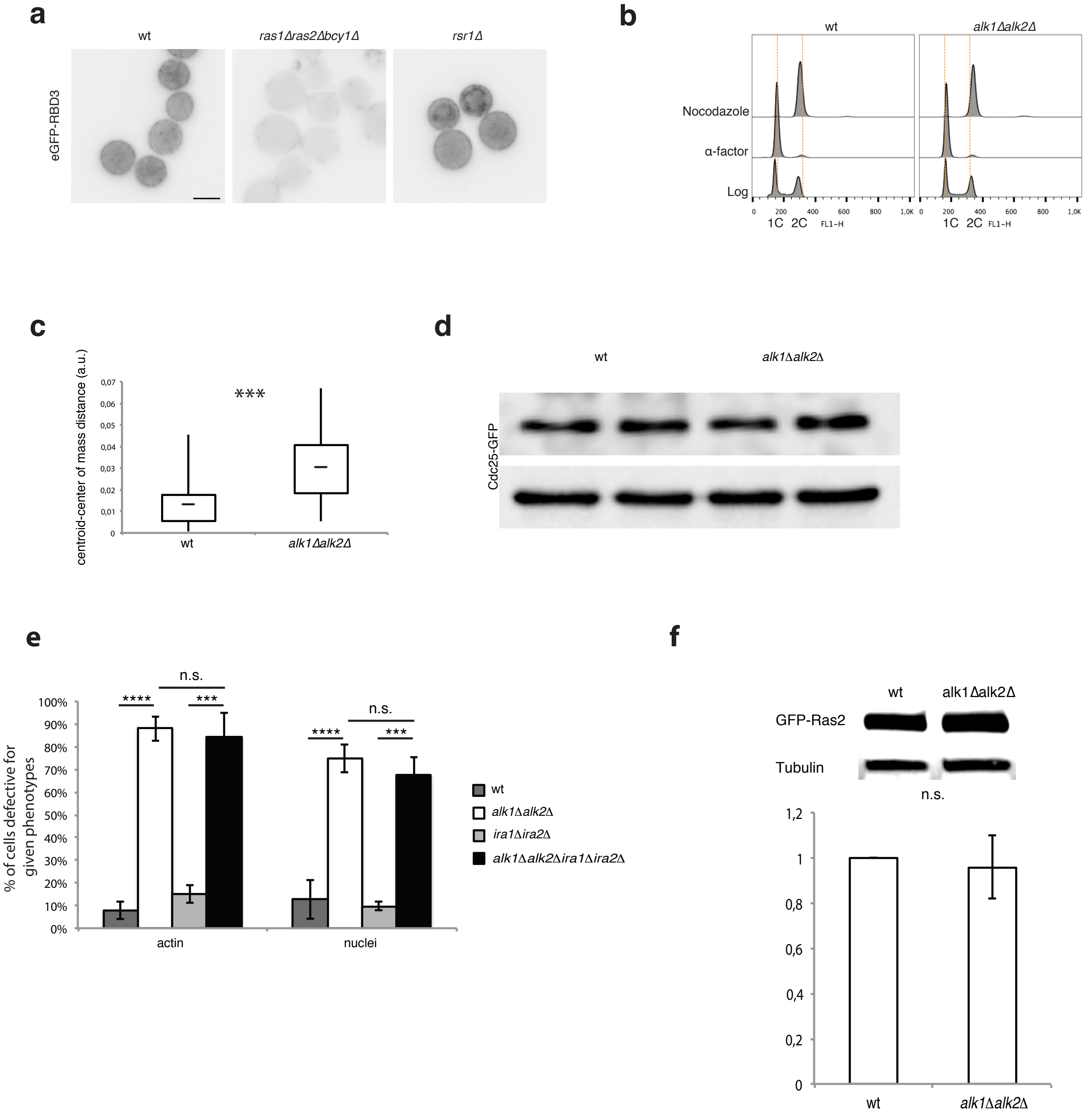
a
wt
ras1Δras2Δbcy1Δ
rsr1Δ
eGFP-RBD3
b
wt
alk1Δalk2Δ
Nocodazole
α-factor
Log
0
200
400
600
800
1,0K
1C
2C
FL1-H
c
centroid-center of mass distance (a.u.)
0,07
0,06
0,05
0,04
0,03
0,02
0,01
0
***
wt
alk1Δalk2Δ
d
wt
alk1Δalk2Δ
Cdc25-GFP
e
% of cells defective for given phenotypes
100%
90%
80%
70%
60%
50%
40%
30%
20%
10%
0%
n.s.
****
***
actin
nuclei
wt
alk1Δalk2Δ
ira1Δira2Δ
alk1Δalk2Δira1Δira2Δ
f
wt
alk1Δalk2Δ
GFP-Ras2
Tubulin
n.s.
1,2
1
0,8
0,6
0,4
0,2
0

Fig S4. Related to Figure 4.

(a) Specificity of the eGFP-RBD3 probe was evaluated comparing the fluorescence signal observed in wt strains, in cells lacking *RAS* or lacking *RSR1*; scale bar: 5µm. Plot (b) shows the cytofluorimetric analysis of the cell cycle arrests for the experiment described in Figs. 4a-b. (c) shows the centroid-center of mass distance, normalized on daughter area and average daughter cell circularity, of 60 cells from 3 independent experiments treated as in Figs. 4a-b. Boxes include 50% of data points, line represents the average distance and whiskers report the minimum and maximum values. (d-e) Cells of the indicated strains were arrested in G1 and then released for 2.5 hours in nocodazole. Panel (d) shows representative images of the localization of Cdc25-GFP in wild-type or haspin-lacking cells. Graph (e) reports the impact of *IRA1* and *IRA2* deletion on actin and nuclear segregation scored by fluorescence microscopy from 3 independent experiments, counting 100 cells per repeat; error bars represent standard deviation. Panel (f) shows a western blot analysis of the expression levels of *GFP-RAS2* for the experiment in Figure 4c-e. Data from 3 independent experiments are plotted in the right panel. Scale bars in a,d: 5µm. Error bars correspond to standard deviation. t-test was applied as a statistical measurement in (c,e,f); n.s.: not significant; * : p-value <0.05; ** : p-value <0.01; *** : p-value <0.005; **** : p-value <0.001.
