# Supplementary material for: Haspin regulates Ras localization to promote Cdc24-driven mitotic depolarization
Source: Cell Discov. 2020 Jun 23;6:42. doi: 10.1038/s41421-020-0170-2 (PMC7308332; doi:10.1038/s41421-020-0170-2)
Supplement: Supplementary file 5 — Supplementary Figure S5 [file 41421_2020_170_MOESM5_ESM.pdf]

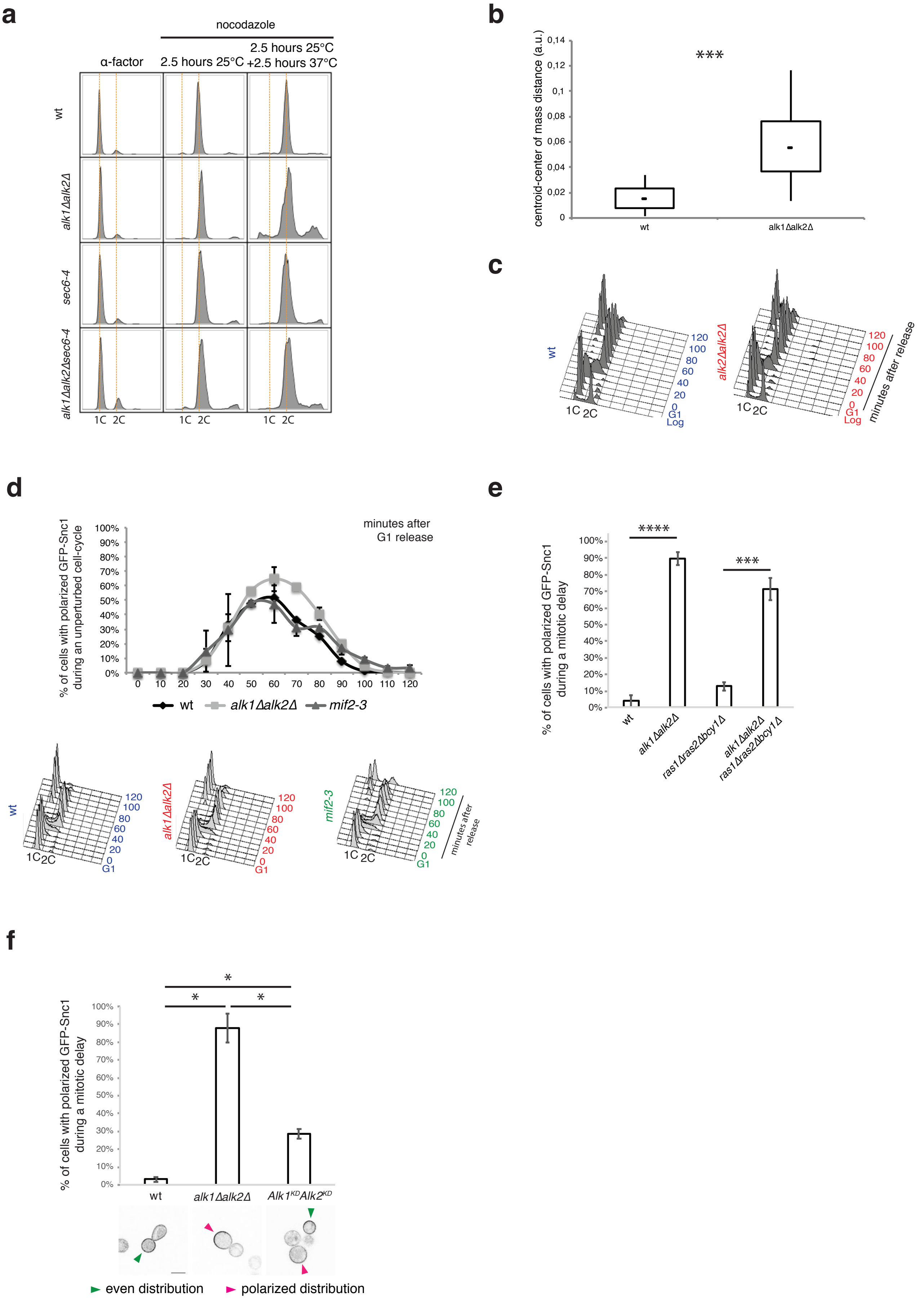
a
nocodazole
α-factor
2.5 hours 25°C
2.5 hours 25°C +2.5 hours 37°C
wt
alk1Δalk2Δ
sec6-4
alk1Δalk2Δsec6-4
1C 2C
b
centroid-center of mass distance (a.u.)
***
wt
alk1Δalk2Δ
c
wt
alk2Δalk2Δ
minutes after release
Log
d
% of cells with polarized GFP-Snc1 during an unperturbed cell-cycle
minutes after G1 release
wt
alk1Δalk2Δ
mif2-3
e
% of cells with polarized GFP-Snc1 during a mitotic delay
****
***
wt
alk1Δalk2Δ
ras1Δras2Δbcy1Δ
alk1Δalk2Δ ras1Δras2Δbcy1Δ
f
% of cells with polarized GFP-Snc1 during a mitotic delay
wt
alk1Δalk2Δ
Alk1KDAlk2KD
even distribution
polarized distribution

Fig S5. Related to Figure 5.

(a) Cell cycle analysis by FACS of experiment in Fig. 5a. The plot in (b) represents the centroid-center of fluorescence mass distance of Snc1-GFP, normalized on the daughter cell area and average daughter cell strain circularity, calculated on 20 cells per strain; boxes include 50% of data points, lines represents the average distance and whiskers report the minimum and maximum values. (c) Cell cycle analysis by FACS of experiment in Fig. 5b and relative quantification (blue: wt; red: *alk1Δalk2Δ*). (d) cells of given strains were synchronized in G1 at 25°C and then released at 33°C, taking samples every 10 minutes to monitor Snc1 distribution by fluorescence microscopy and cell cycle progression by FACS (for quantification: blue: wt; red: *alk1Δalk2Δ*, green: *mif2-3*; The data derive from two independent experiments, counting 100 cells per repeat). (e) Cells of the indicated strains, expressing GFP-Snc1, were treated with nocodazole for 3 hours. Samples were then analyzed to monitor distribution of the SNARE. The panel reports the percentage of cells with polarized Snc1, calculated in 3 independent experiments, counting 100 cells per repeat. (f) Cells of given strains were synchronized in G1 and then released for 2.5 hours in nocodazole-containing medium. At the end of the treatment cells were fixed and analyzed by fluorescence microscopy. The graph shows the percentage of cells exhibiting polarized Snc1-GFP, calculated in 2 independent experiments, counting 100 cells per repeat. Green and magenta arrows point to cells with even or polarized Snc1-GFP signal, respectively. t-test was applied as a statistical measurement in (b,d,e,f); n.s.: not significant; * : p-value <0.05; ** : p-value <0.01; *** : p-value <0.005; **** : p-value <0.001.
