# Supplementary material for: Haspin regulates Ras localization to promote Cdc24-driven mitotic depolarization
Source: Cell Discov. 2020 Jun 23;6:42. doi: 10.1038/s41421-020-0170-2 (PMC7308332; doi:10.1038/s41421-020-0170-2)
Supplement: Supplementary file 6 — Supplementary Figure S6 [file 41421_2020_170_MOESM6_ESM.pdf]

a

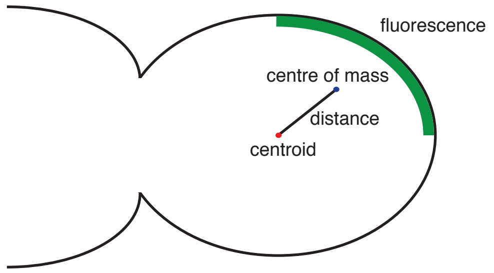

b

$$\bar{I}_i = \bar{I}_{m+1-i} = \frac{\sum_{j=1}^{n} \frac{I_{i,j}+I_{m+1-i,j}}{2\sum_{i=1}^{m} I_{i,j}}}{n}$$

c

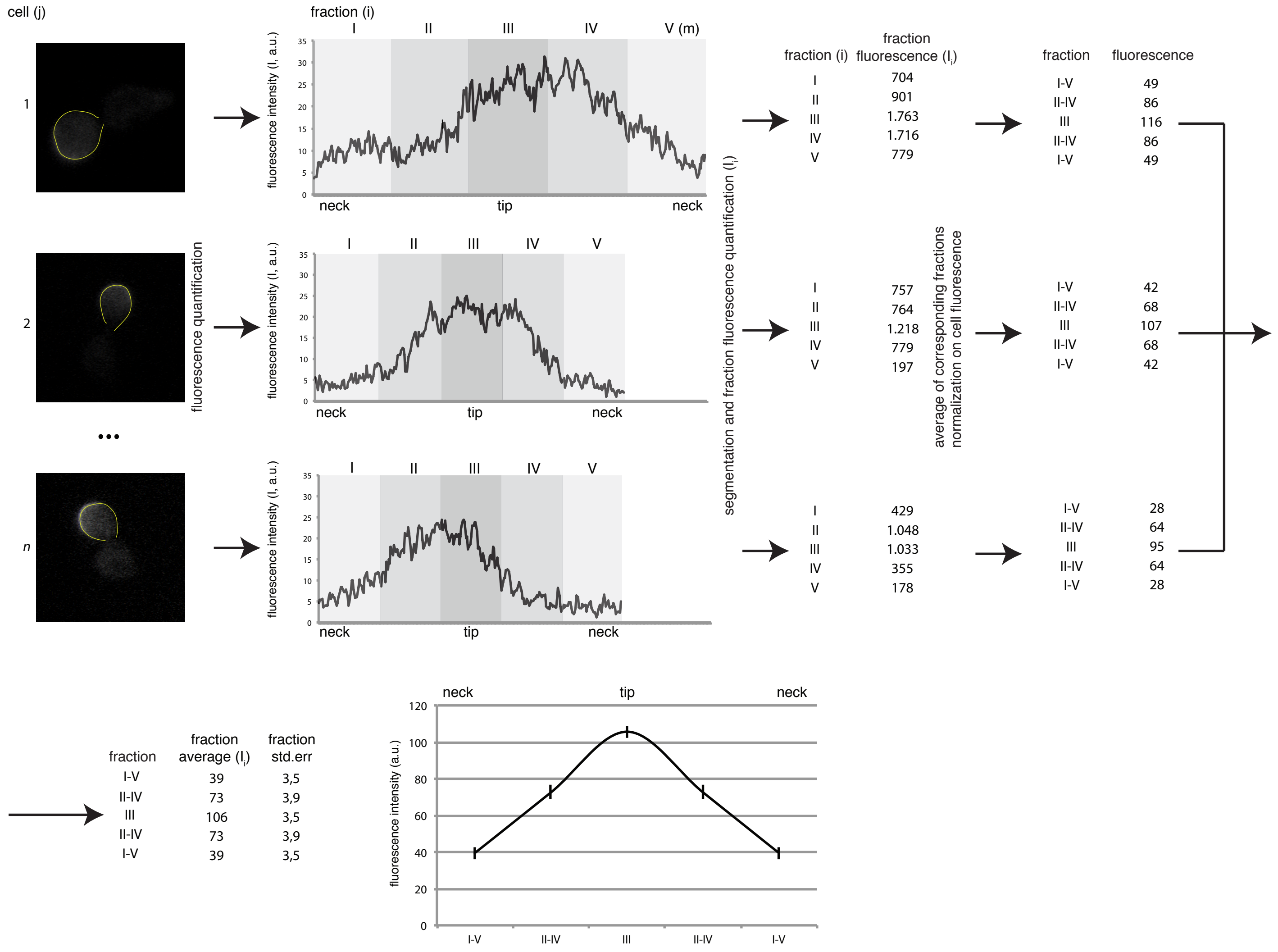

Fig S6. Fluorescence quantifications.

Figure (a) schematically represents the centroid-center of mass distance. This value was then normalized according to cell dimension and circularity. The average intensity of a given fraction was determined using the equation in panel (b), in which I represents the intensity, i a fraction, j a cell, n the total number of cells (in our analysis 60) and m the total number of sections (in our analysis 100). Panel (c) shows an application of equation b in which n=3 and m=5.
